# Psychedelic Drugs or Hallucinogens: Exploring Their Medicinal Potential

**DOI:** 10.7759/cureus.48719

**Published:** 2023-11-13

**Authors:** Priyanshu Raj, Shyambabu Rauniyar, Bhagyesh Sapkale

**Affiliations:** 1 Medicine, Jawaharlal Nehru Medical College, Datta Meghe Institute of Higher Education and Research, Wardha, IND; 2 Anatomy, Jawaharlal Nehru Medical College, Datta Meghe Institute of Higher Education and Research, Wardha, IND

**Keywords:** psychedelic medicines, hallucinogens uses, psychedelic drug research, psychedelic drugs, hallucinogens

## Abstract

Serotonergic hallucinogens also referred to as psychedelics, are psychoactive substances that profoundly alter perception, mood, and cognitive processes. These substances, historically intertwined with religious and cultural rituals, offer profound effects that extend beyond mere hallucinations to profoundly altered states of consciousness. Notable compounds like Lysergic acid diethylamide (LSD) and psilocybin, potent in their action on serotonin receptors, play pivotal roles in influencing brain functions. Despite societal misconceptions that have overshadowed their potential, contemporary research increasingly recognizes their therapeutic value. These substances have shown promise in treating neuropsychiatric disorders such as depression, post-traumatic stress disorder (PTSD), and anxiety, leveraging their influence on neuroplasticity. Furthermore, they exhibit therapeutic potential across various conditions, challenging conventional treatment methodologies. Compared to substances like alcohol, traditional psychedelics like LSD and psilocybin emerge as relatively safer substances. The modern revival of scientific interest in psychedelics necessitates a renewed perspective, viewing them not just as recreational entities but as potent therapeutic tools. Harnessing their actual value mandates rigorous scientific investigations and a receptive societal discourse. A re-evaluation of their classification following international criteria is necessary in light of this increasing understanding. Hallucinogens or psychedelic drugs, if used correctly, can potentially be potential treatments for mental illness, signalling a paradigm shift from traditional techniques. To dispel myths and use their therapeutic advantages, embracing this potential necessitates thorough scientific investigation together with an open societal discourse.

## Introduction and background

Psychedelic medications are a class of chemicals that alter or improve sensory perceptions, mental processes, and vigour. Mescaline, psilocybin, dimethyltryptamine (DMT), and d-lysergic acid dimethylamine (LSD) are examples of traditional psychedelic drugs. Natural psychedelics, such as psilocybin mushrooms and peyote, come from naturally occurring plants and fungi, have been used for a very long time, including a variety of chemicals, and provide a more "earthy" and varied experience. Conversely, synthetic psychedelics LSD and 3,4-Methyl​enedioxy methamphetamine (MDMA) are relatively new innovations that have been chemically manufactured and refined to concentrate on the main hallucinogenic ingredient and have predictable effects. While synthetic psychedelics are linked to contemporary counterculture and scientific study, natural psychedelics are frequently associated with cultural and historical value. They may be subject to seasonal availability and regulatory limits. Studies investigate the possible therapeutic benefits, considering their unique attributes and practical uses. A class of chemicals known as hallucinogens or psychedelics alters a person's view of reality. They alter a person's ideas and emotions as well. LSD, peyote, phenylcyclohexyl piperidine (PCP), psilocybin, and other substances fall under the category of hallucinogens [1]. The term "psychedelic," which was first used by psychiatrist Humphrey Osmond in a 1956 letter to Aldous Huxley, comes from the ancient Greek terms "psyche" (meaning "mind" or "soul") and "dēlein" (meaning "to reveal") [2]. The primary effects of psychedelics, a type of hallucinogenic drug, are the start of unexpected mental states (often called "trips" or "psychedelic experiences") and an apparent extension of awareness. They are referred to as "classic hallucinogens" at times, "serotonergic hallucinogens," or "serotonergic psychedelics," and "psychedelic" is occasionally used in a more general sense to refer to a variety of hallucinogens [3]. Psilocybin and other psychedelics derived from plants have long been employed therapeutically. After the first English-language study on LSD was published in 1950, psychedelics briefly became associated with psychology and psychiatry. Before prohibitive legislation in the middle of the 1960s virtually put a stop to all large psychedelic research programs, drugs like LSD first had therapeutic potential. The most common uses of these drugs in psychotherapy were to treat alcoholism and mood disorders [4].

Synthetic LSD, as well as psilocybin from magic mushrooms, N,N-dimethyltryptamine (DMT), which comes from the herbal beverage ayahuasca, and mescaline from the Peyote cactus, are examples of traditional psychedelic chemicals. They are all agonists of the 5-hydroxytryptamine (HT) 2A receptor, which activates serotonin receptors [5]. According to a list of 20 legal and illegal substances and their negative impacts on users and society, psilocybin and LSD were among the least dangerous.

Using LSD recreationally has been linked to a syndrome known as hallucinogen-persisting perception disorder (HPPD) [6]. The United Nations classifies psychedelic drugs as having a high potential for abuse, substantial negative impacts on public health, and no possibility for medicinal use. However, this classification has come under fire [7]. An American study involving 130,000 people found no link between using psychedelics (LSD, psilocybin, or mescaline) and mental health issues. Of these participants, 13.4% acknowledged using them currently or in the past [8]. The effects of classic psychedelic drugs include derealization, depersonalization, altered time perception, and audiovisual synesthesia [9]. Users of traditional psychedelic drugs had a lower risk of mental illnesses, including suicidal ideation.

In contrast, users of other illegal substances, such as cocaine, amphetamines, opioids, and synthetic drugs like MDMA (Ecstasy), had a higher risk [10]. In cancer patients, anxiety and sadness worsen morbidity and speed up death. The existing therapy choices are frequently ineffective [11].

## Review

Search methodology

**Figure 1:**
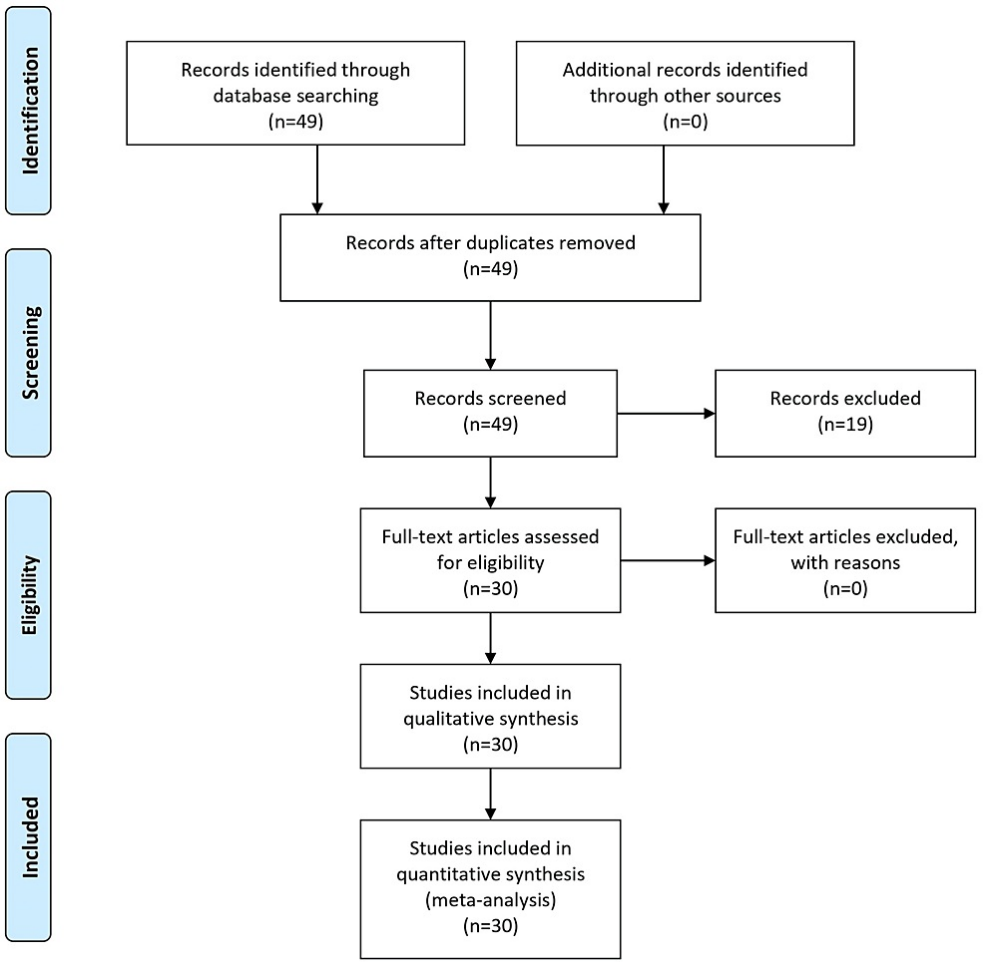
PRISMA flow chart diagram PRISMA: Preferred Reporting Items for Systematic Reviews and Meta-Analyses

To compile a comprehensive review of serotonergic hallucinogens and their potential therapeutic applications, a systematic search methodology was adopted. Primary databases like PubMed, Google Scholar, PsycINFO, Web of Science, and the Cochrane Library were scoured using keywords such as Psychedelics, LSD, Psilocybin, Serotonin receptors, and more, targeting publications from 2000-2023 in the English language. Emphasis was placed on peer-reviewed articles, clinical trials, and meta-analyses. Inclusion criteria focused on studies that delved into the therapeutic potential and mechanism of action of these hallucinogens or examined their effects on neuropsychiatric disorders. Exclusions comprised studies outside the set timeframe, non-peer-reviewed publications, and articles solely focused on recreational use. Once relevant articles were identified, data such as author names, publication year, study design, findings, and therapeutic applications were meticulously extracted. This gathered information was methodically arranged to gain insights into the historical context, primary effects, therapeutic potential, and safety profiles of the hallucinogens. Any overlapping or duplicate studies were filtered out to maintain the review's integrity. To ensure organized citation and easy reference access, all sources were managed using the Zotero reference management software (www.zotero.org). This rigorous methodology assured an all-encompassing exploration of the medicinal potential of serotonergic hallucinogens, leading to an informed discussion on their place in contemporary medicine. Figure 1 shows the Preferred Reporting Items for Systematic Reviews and Meta-Analyses (PRISMA) flow diagram for the literature search.

Psychedelics or hallucinogens

Serotonergic hallucinogens, also called psychedelics, are highly affecting psychoactive materials that affect emotion, perception, and several other related processes [12]. They often don't cause dependency or addiction and are thought to be physically safe. Their history predates recorded history, and early cultures used them in various societal and ritual situations. Since psychedelic substances did not offer very accurate representations of psychosis or mental illness, it became more appropriate to refer to them as hallucinogens, which is another derogatory term implying that they primarily cause hallucinations. Although it is still commonly used and appears to still be the favoured nomenclature for these medications in most scientific writing, that is not what they do in the majority of users at typical doses. Additionally, the term "hallucinogen" is frequently used to refer to a broad range of psychoactive substances, including cannabis, "ecstasy," dissociative agents, and other substances. D-lysergic acid diethylamide, also known as LSD-25, was created in 1938 by a chemist working for Sandoz Laboratories in Switzerland and is one of the most famous psychedelic substances. When compared to other drugs like mescaline, psilocin, and psilocybin, LSD was shown to be hundreds or thousands of times more potent [13]. The structure-activity connections of hallucinogens and the subsequent signalling processes responsible for mediating their effects have also been extensively studied in animal models. They have also aided in clarifying how various receptors and transporters control how hallucinogen effects are expressed [14]. Because of their unique capacity to alter perceptual states, psychedelic substances are adequate resources for studying how the mind works. Additionally, psychedelics are chemicals that may help people with a variety of neuropsychiatric conditions, such as depression, anxiety, and substance use disorders [15]. Various plants that contain naturally occurring psychedelics, such as the mescaline-containing cactus Lophophora williamsii, the psilocybe species of mushrooms, and the lysergamide-containing seeds of "Morning Glories" (Ipomoea species), have reportedly been used for millennia in shamanic and religious rituals (Nichols, 2016).

**Figure 2:**
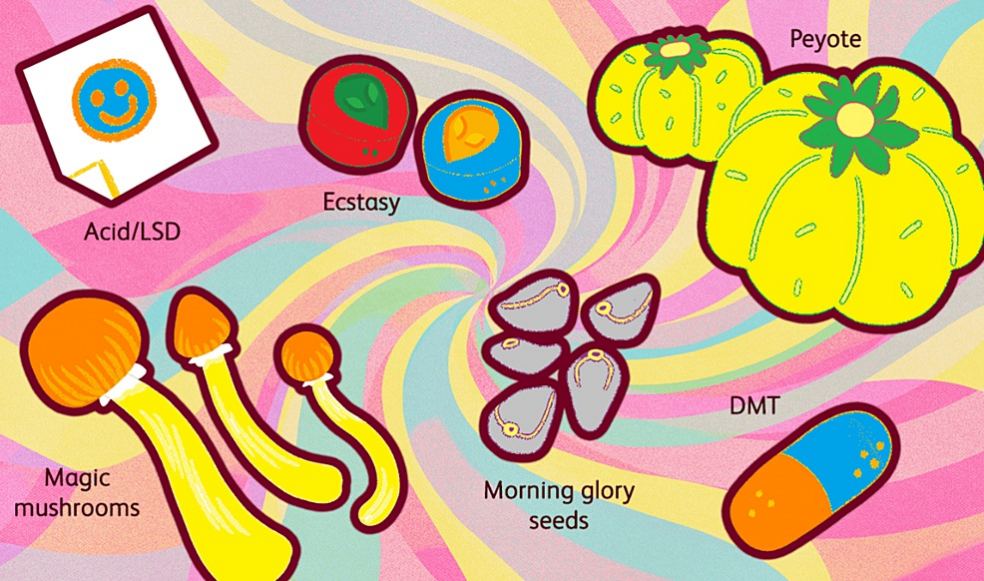
Types of psychedelic drugs Lysergic acid diethylamide (LSD), Magic Mushrooms, Dimethyltryptamine (DMT), Peyote, Ecstasy, Morning Glory Seeds Source: Reference no. [17]

Since the 1950s and 1960s, semi-synthetic hallucinogens like lysergic acid diethylamide (LSD) and substituted amphetamines like 2,5-dimethoxy-4-methylamphetamine (LSD) have been available for purchase and used recreationally [16]. LSD increases emotional empathy while decreasing the left amygdala response to frightened stimuli and impairing the perception of sad and fearful faces. It also enhances the emotional reaction to and significance of music, so overall Intimacy, openness, trust, and suggestibility were all elevated by LSD [17]. Sensorimotor gating impairments were acutely generated by LSD, which is consistent with schizophrenia [17]. Figure 2 shows types of psychedelic drugs.

LSD (Lysergic acid diethylamide): A potent synthetic psychedelic that produces alterations in perception, mood, and various cognitive processes. It was first synthesized in 1938 from a compound found in the ergot fungus. Magic Mushrooms: A naturally occurring psychedelic found in certain mushrooms, often called "magic mushrooms" or "shrooms". The active compound is converted in the body to psilocybin, which produces mind-altering effects.

DMT (Dimethyltryptamine): A powerful psychedelic found in many plants and animals and is also produced endogenously in the human body. It can produce highly intense and short-lasting effects when smoked.

Peyote: A naturally occurring psychedelic found in several cactus species, most notably the peyote cactus. It produces visual hallucinations and deep introspection.

Ecstasy: While it's primarily classified as an empathogen or entactogen, it has psychedelic properties. It's known for inducing love, empathy, and connection with others and is frequently referred to as "Ecstasy" or "Molly" in its street form. However, these may be adulterated with other substances.

Morning Glory Seeds: A plant of seeds native to Oaxaca, Mexico, that, when its seeds are used, produces short, intense, and unique psychedelic experiences.

Beyond PTSD and depression, 3,4-methyl​enedioxy​methamphetamine-assisted psychotherapy (MAP)and psilocybin-assisted psychotherapy (PAP) have demonstrated transdiagnostic therapeutic promise, particularly as therapies for anxiety disorders [18]. Hallucinogens can be categorized into various groups using a broad definition based on chemical structure and pharmacological mechanism of action. Psychedelics, entactogens, dissociatives, and other unusual hallucinogens are some of them [19]. There are some significant similarities between these classes even though they do not share a common underlying mechanism of action, as evidenced by their capacity to elicit brief but significant shifts in consciousness that involve sudden changes in physical, perceptual, cognitive, and affective processes. Religious groups have always used psychedelics for ceremonial purposes. American researchers first became aware of the Psilocybe mexicana mushroom's hallucinogenic qualities through one of these customary Mazatec rituals, the velada [20]. Depression, post-traumatic stress disorder (PTSD), and substance use disorders are just a few of the many brain problems that psychedelics may be able to help [21]. Psychedelics may be able to prevent cortical atrophy, a symptom of many neuropsychiatric and neurodegenerative illnesses, by influencing the expression of neurotrophic factors, turning on neuronal growth and survival processes, and modifying the immune system [21].

Psychedelics drugs action

The 5-HT2A receptor is the primary target location of hallucinogens, is essential for the health of corticoneurons and is not regulated in diseases like Alzheimer's. While more research is required to examine the effects of psychedelics in animal models of neurodegenerative disorders, it is evident that more research is necessary, given the substantial impacts of these substances on inflammation and structural and functional neuroplasticity. The hallucinations tend to change the subject's entire world at higher doses (particularly in the case of DMT, LSD, 5-methoxy-N,N-dimethyltryptamine (5-MeO-DMT), or salvinorin-A) [22]. DMT or salvinorin-A users often humorously allude to "the real world" as though they had travelled to unexplored territory or another planet. Because of this, experiences induced by high doses of hallucinogenic drugs should not be interpreted as mere perceptions of external objects or voices surrounded by an unaffected reality, but rather as powerful hallucinations involving a transformation of the person's entire subjective reality and thought patterns through which "reality" is typically constructed or perceived. Entheogens, which include psilocybin and LSD, are known to induce introspection, fresh insights, perspective changes, and a reorientation of one's understanding of one's past, present, and future. As an empathogen, 3,4-methyl​enedioxy​methamphetamine (MDMA) is said to have strong emotional effects on friendliness, empathy, and openness. These characteristics could be the reason why MDMA is useful in therapy for those with severe PTSD [23]. LSD's D-isomer alone was discovered to have hallucinogenic properties. It was believed that serotonin, a crucial brain amine, was negatively affected by substances including LSD, psilocybin, psilocin, bufotenine, and harmine. Contradictory evidence existed, nevertheless, to support this. It was discovered that some substances that prevented the brain's serotonin receptors from functioning had no psychedelic effect. It was found that mescaline shares structural similarities with the catecholamines adrenaline and norepinephrine, which are released by the adrenal glands and are thought to function as neurotransmitters in the central nervous system [24].

Psychedelics effects

A kaleidoscope of visual patterns is produced by the perception-altering drug LSD. While the effects of LSD can occasionally appear real, the majority of users are aware that these are illusions only. Ecstasy elevates mood and promotes feelings of empathy and connection [25]. The ketamine-induced out-of-body experience can be exciting or soothing. Intense, brief psychedelic experiences, such as seeing colours or hearing noises, are produced by salvia. As a class of drugs, hallucinogens are typically accepted to include a wide variety of pharmacological substances, with mechanisms of action including cannabinoid agonism, NMDA antagonism, muscarinic receptor antagonism, and opioid agonis [26]. Based on general substance use dependency criteria, many of which do not apply to psychedelic drugs, The category of hallucinogen dependence is different from HUD. For hallucinogens, several withdrawal symptoms and signs are unknown. Compared to hallucinogen dependence, hallucinogen misuse involves far less frequent usage of the drugs [27]. Psychedelics from historical rituals to therapeutic potential are explained in Table 1.

**Table 1:** Psychedelics: from historical rituals to therapeutic potential This is an overview of what psychedelics are. They are termed serotonergic hallucinogens, which means they interact primarily with the serotonin system in the brain, affecting various mental processes like perceptions, moods, and cognitive functioning. Source: Reference nos. [26,27]

Category	Details/Examples
Definition	Serotonergic hallucinogens that influence perceptions, mood, and cognitive processes.
Historical Context	Intertwined with religious and cultural rituals.
Common Misconceptions	Seen as only causing hallucinations; often perceived as mere recreational agents.
Actual Effects	Immersion into altered states of consciousness beyond mere hallucinations.
Prominent Substances	LSD and Psilocybin (known for potency and action on serotonin receptors).
Therapeutic Potential	Neuropsychiatric disorders (Depression, PTSD, Anxiety); Potential against neurodegenerative conditions; Transdiagnostic therapeutic possibilities.
Mechanism of Action	Influence on neuroplasticity and serotonin receptors.
Safety & Risk	Low addiction risk, especially when compared to substances like alcohol.
International Classification	Current classification, typically in contradiction with their safety profile; demands reconsideration.
Current Perspective	Shift from recreational agents to potential therapeutic tools.
Future Implications	Potential breakthroughs in mental health treatments under appropriate clinical conditions.
Requirements for Progress	Rigorous scientific inquiry and open societal dialogue.

Psychedelics, historically rooted in spiritual rituals, have always been a subject of both reverence and controversy. At their core, psychedelics, or serotonergic hallucinogens, are substances that primarily interact with the serotonin system in the brain [12]. This interaction profoundly affects various mental processes, influencing perceptions, moods, and cognitive functioning. Over the centuries, many cultures and societies have incorporated these substances into religious and spiritual ceremonies. They valued the ability of these drugs to invoke mystical experiences and profound alterations in consciousness [16]. However, as time progressed and the world changed, the modern perception of these compounds shifted. There's a prevalent misconception today that associates these substances solely with hallucinations or dismisses them as mere recreational drugs. Such views often overlook the profound introspective, emotionally cathartic, and perspective-shifting experiences they can induce. Beyond the mere sensory hallucinations, psychedelics immerse users in profoundly altered states of consciousness that can be profoundly transformative [17].

Psychedelics and neuro-pharmaceutical treatments

When discussing these substances, two names often come to the fore: LSD and psilocybin. Both have been extensively researched and are mainly known for their potent effects on serotonin receptors in the brain. Their potential, however, goes beyond recreational use. Contemporary research has illuminated their therapeutic potential, suggesting they could be pivotal in addressing neuropsychiatric ailments like depression, PTSD, and anxiety. These findings hint at the promise these substances hold in possibly treating even neurodegenerative conditions. Their influence on neuroplasticity, the brain's innate ability to adapt and form new neural connections, is central to their mechanism of action, marking a departure from the effects of more conventional treatments. In the broader landscape of drugs and substances, traditional psychedelics like LSD and psilocybin stand out for their relatively benign risk profile, especially when juxtaposed against legal and widely accepted substances like alcohol. Yet, the international stance on these substances remains stringent. Their classification often hampers research and restricts therapeutic access despite their notable safety profile. This restrictive view is, however, undergoing a significant transformation. A growing consensus in the scientific community acknowledges the therapeutic potential of psychedelics, challenging their reputation as mere recreational entities. Looking ahead, as scientific inquiry deepens, there's palpable optimism surrounding psychedelics. The hope is that with continued research, these substances could revolutionize mental health treatments. However, to harness their full potential, they must be administered under controlled conditions with the requisite expertise. Progress in this field mandates rigorous scientific scrutiny and an open societal discourse.

Dispelling entrenched misconceptions and fostering a well-informed perspective will be pivotal in realizing the therapeutic promise these ancient substances hold for modern medicine. Lifetime exposure to psychedelics was favourably correlated with openness scores. Among all recreational users of MDMA and psychedelics, frontal serotonin transporter, but not frontal serotonin 2A receptor (5-HT2AR) binding, was strongly correlated with openness scores [28]. Potent serotonergic agonists that bind to the majority of serotonin receptors are LSD and psilocybin. Since 5-HT2A antagonists prevent these effects, their psychedelic effects are most likely the result of actions at 5-HT2A receptors [29]. LSD also increases dopamine D2 receptor activity, which may help explain why it has psychotomimetic effects, in contrast to the majority of other medications in its class. Because of their unfavourable side effects, traditional antidepressants and psychotic medications are becoming less popular [30]. With proper medical supervision, psychedelic neuro-pharmaceutical treatments could be a therapy option for common mental conditions, including anxiety and depression, as well as a viable alternative.

## Conclusions

The profound exploration into serotonergic hallucinogens, commonly termed psychedelics, underscores their potential in reshaping perceptions, mood, and cognitive processes. Historically, these substances have been intertwined with religious and cultural rituals. Their effects, which are often misconstrued, go beyond mere hallucinations; in actuality, they immerse users into altered states of consciousness. Notably, substances like LSD and psilocybin stand out for their potency and significant action on the serotonin receptors, a primary factor in their modulatory effects on the human brain. Interestingly, the general misconception surrounding these compounds, primarily driven by societal views, overshadows their therapeutic potential. Contemporary research suggests potential applications for these substances in addressing neuropsychiatric disorders, including depression, PTSD, and anxiety. Their mode of action, particularly their influence on neuroplasticity, positions them as promising agents against neurodegenerative conditions. Moreover, they display transdiagnostic therapeutic possibilities, transcending the limitations of conventional treatments. In the grand tapestry of drug use, traditional psychedelics like LSD and psilocybin emerge as relatively safe. The low addiction risk they pose, juxtaposed against the societal and individual harms of drugs like alcohol, challenges established narratives and demands reconsideration of international classification protocols. Additionally, the exploration into psychedelics underscores a fundamental shift from understanding these substances as mere recreational agents to potential therapeutic tools. In essence, the resurgence of interest in psychedelics in the scientific community emphasizes the need to approach them with a fresh perspective. These substances, when administered under appropriate clinical conditions, could offer breakthroughs in mental health treatments, signalling a paradigm shift from conventional modalities. Embracing their potential requires rigorous scientific inquiry and open societal dialogue, dispelling misconceptions and harnessing their therapeutic value.
